# Maternal postnatal depression predicts altered offspring biological stress reactivity in adulthood

**DOI:** 10.1016/j.psyneuen.2014.12.003

**Published:** 2015-02

**Authors:** Tom J. Barry, Lynne Murray, R.M. Pasco Fearon, Christina Moutsiana, Peter Cooper, Ian M. Goodyer, Joe Herbert, Sarah L. Halligan

**Affiliations:** a Centre for the Psychology of Learning and Experimental Psychopathology, University of Leuven, Tiensestraat 102, Leuven 3000, Belgium; b School of Psychology and CLS, University of Reading, Reading RG6 6AL, UK; c Department of Psychology, Stellenbosch University, Stellenbosch, South Africa; d Research Department of Clinical, Educational and Health Psychology, University College London, 1-19 Torrington Place, London WC1E 7HB, UK; e Experimental Psychology, University College London, Gower Street, London WC1E 6BT, UK; f Department of Psychiatry, Cambridge University, Cambridge CB2 2AH, UK; g John van Geest Centre for Brain Repair, Department of Clinical Neuroscience, Cambridge University, Cambridge CB2 3BY, UK; h Department of Psychology, University of Bath, Bath BA2 7AY, UK

**Keywords:** Depression, Stress sensitivity, Cortisol, Maternal depression, Longitudinal, Hypothalamic–pituitary–adrenal axis

## Abstract

The offspring of depressed parents have been found to show elevated basal levels of the stress hormone cortisol. Whether heightened cortisol stress reactivity is also present in this group has yet to be clearly demonstrated. We tested whether postnatal maternal depression predicts subsequent increases in offspring biological sensitivity to social stress, as indexed by elevated cortisol reactivity.

Participants (mean age 22.4-years) derived from a 22-year prospective longitudinal study of the offspring of mothers who had postnatal depression (PND group; *n* = 38) and a control group (*n* = 38). Salivary cortisol response to a social-evaluative threat (Trier Social Stress Test) was measured.

Hierarchical linear modelling indicated that PND group offspring showed greater cortisol reactivity to the stress test than control group participants. Group differences were not explained by offspring depressive or anxiety symptoms, experiences of negative life events, elevated basal cortisol at age 13-years, subsequent exposure to maternal depression, or other key covariates.

The findings indicate that the presence of early maternal depression can predict offspring biological sensitivity to social stress in adulthood, with potential implications for broader functioning.

## Highlights

• Postnatally depressed (PND) mothers’ offspring showed increased cortisol stress reactivity.
• This response at age 22-years was relative to control offspring.
• There was also evidence of *stable* alterations in cortisol secretion in the PND group.
• Offspring or subsequent maternal depression did not explain group differences.
• Early maternal depression can predict offspring biological sensitivity to stress.

## 1 Introduction

Research has established that the offspring of depressed parents are themselves at elevated risk of depressive disorder by early adolescence (Halligan et al., 2007b; Murray et al., 2011; Hammen et al., 2012). Efforts to understand the biological mechanisms by which such intergenerational risks are conferred have included a focus on the hypothalamic–pituitary–adrenal (HPA) axis. The HPA axis is fundamental to human stress responding, and HPA disturbances have been identified in association with numerous stress-related psychopathologies, most particularly depression (Gold et al., 1988). Basal cortisol concentrations, which are regulated by the HPA axis, may be elevated in children and adolescents during a depressive episode (Lopez-Duran et al., 2009), as well as in adults (Gold et al., 2002; Pariante and Lightman, 2008; Knorr et al., 2010).

Given the assumed role of HPA axis dysfunction in depressive disorder, a number of studies have examined basal cortisol secretion in at-risk groups, including the offspring of depressed parents. Elevated basal cortisol levels have been demonstrated in association with the presence of parental depression across a number of studies (Essex et al., 2002; Halligan et al., 2004; Ellenbogen et al., 2006; Mannie et al., 2007). Higher basal cortisol levels have, in turn, been found to predict subsequent depressive symptom levels (Halligan et al., 2007a; Ellenbogen et al., 2011), although there is contradictory evidence on this point (Carnegie et al., 2014). Longitudinal studies have tentatively indicated that the presence of maternal depression early in development may be particularly important (Essex et al., 2002; Halligan et al., 2004), possibly due to early parenting effects (Murray et al., 2010). Although further evidence is needed, such observations are consistent with rodent models of HPA development which identify the quality of early environmental stimulation as a key determinant of offspring stress reactivity (Meaney et al., 2007).

There is also some evidence of alterations in cortisol stress *reactivity* in the offspring of depressed parents, at least as tested in young infants and children. Elevated cortisol reactivity is suggested by cross-sectional studies of young infants (Azar et al., 2007; Brennan et al., 2008; Waters et al., 2013) and preschool-aged children (Dougherty et al., 2011, 2013) whose parents reported having previous or current depression. These studies have each found relatively higher cortisol levels following a mild stressor in at-risk versus control group infants, but interpretation is complicated by the fact that the provocations used typically did not result in a clear cortisol stress response. Studies where a cortisol stress response was clearly present have not always demonstrated consistent effects. Feldman and colleagues reported that, relative to controls, infants of depressed mothers showed higher cortisol levels overall during a stressor paradigm, but not enhanced reactivity to the stressor (Feldman et al., 2009). In addition, a longitudinal study using immunisation stress in 2 month old infants found antenatal maternal depressive symptoms to be predictive of cortisol reactivity, but the relationship was complex, with both low and high levels of depressive symptoms predicting greater reactivity (Fernandes et al., 2014). In contrast to studies of infants and young children, investigations of older samples have the advantage that standard paradigms exist which reliably provoke a cortisol stress response (Kirschbaum et al., 1993), although few studies have examined cortisol reactivity in youth or adults at risk for depression. One study examining the at-risk offspring of parents with bipolar disorder did not find cortisol responses to stress to distinguish them from controls (Ellenbogen et al., 2006). Moreover, in contrast to the pattern of findings deriving from infants and young children, a second study examining parental retrospective self-reports of “depressive problems” found that these were associated with *reduced* cortisol stress reactivity in adolescent girls, and were not associated with cortisol responses in boys (Bouma et al., 2011).

The limited availability of robust evidence for enhanced cortisol reactivity to stress in the offspring of depressed parents is significant, as elevations in basal cortisol levels that have been observed in this group have been proposed to reflect HPA axis dysregulation linked to increased vulnerability to stressors (Ostiguy et al., 2011). Further investigation is warranted. Here, we report on cortisol stress reactivity in a longitudinally studied sample of young adults. Participants in the study were recruited shortly following birth based on the presence or absence of maternal depression during the postnatal period; and maternal depression was repeatedly assessed throughout the 22-years of the study, providing a clear profile of offspring exposure to depressive disorder. In the same sample, we previously identified elevations in basal morning cortisol secretion in the 13-year old offspring of mothers who experienced postnatal depression (PND) versus control group offspring (Halligan et al., 2004). In the current study, offspring cortisol reactivity in response to a standard social stressor (Trier Social Stress Test: TSST) was measured at age 22-years.

We used hierarchical linear modelling (HLM) to model changes in cortisol concentrations over the course of the TSST in relation to PND group status, and to test the hypothesis that PND versus control group offspring would show enhanced cortisol stress reactivity as young adults. We controlled for gender, history of offspring depressive or anxiety disorder, current offspring depressive or anxiety symptoms, and incidences of negative life events in our analyses, given their potential relationship with cortisol reactivity (Burke et al., 2005; Petrowski et al., 2010; Allen et al., 2014). Since elevated basal cortisol concentrations have previously been interpreted as a marker for HPA dysregulation or increased reactivity to daily stress (Goodyer et al., 2000; Kunz-Ebrecht et al., 2004; Ostiguy et al., 2011), we also specifically tested whether basal morning cortisol levels at 13-years predicted the TSST reactivity profile at 22-years. Finally, the assessment of both postnatal and subsequent maternal depression afforded a preliminary investigation of whether there is any evidence for a particular, persistent impact of the presence of maternal depression during early life, as would be predicted by animal models of HPA axis development.

## 2 Materials and methods

Procedures were approved by the University of Reading and the National Health Service Research Ethics Committees. Participants provided written informed consent prior to taking part.

### 2.1 Participants

Participants were part of a prospective longitudinal study of the development of children of postnatally depressed and well women (Murray, 1992). Participants were originally recruited through screening a community sample of primiparous mothers of healthy, full-term infants for PND, by administering the Edinburgh Postnatal Depression Scale (EPDS) (Cox et al., 1987) at 6-weeks postpartum. Women scoring over 12 on the EPDS were interviewed; 61 women who met Research Diagnostic Criteria (Spitzer et al., 1978) for depressive disorder were identified, 58 of whom were recruited for the study. Forty-two non-depressed mothers randomly selected from the same postnatal population were also recruited. The sample previously completed assessments postnatally, and at ages 1.5-years, 5-years, 8.5-years, 13 and 16-years. The current phase of the research took place at mean age 22-years. Seventy-six offspring were able to visit the University of Reading and participate in the laboratory stress test (i.e. 76% of the original sample); 38 offspring of PND mothers (70% of the original PND group) and 38 controls (91% of the original control group) (see Table 1 for participant characteristics^1^), which was a significant difference, *χ*^2^ = 6.19, df = 1, *p* = .013. This was explored further by conducting comparisons of those who did versus did not complete the TSST based on gender, and on key information available from the previous, 16 year assessment; participant history of depression, anxiety, and negative life events; and maternal total study months of depression. There were no significant differences (all *p* > .14) with one exception: those who did not complete the TSST at 22-years had been exposed to more study months of maternal depression, *F* = 9.64, df = 1, 91, *p* = .003. Given that this variable is highly confounded with group status, we also examined whether attrition *within* the PND group was related to duration of maternal depression. Results demonstrated a trend to this effect, *t* = 1.77, df = 51, *p*=.083: PND group participants who did not complete the TSST tended to have mothers who had more months depression (*M* = 32.2, *SD* = 24.3) than those who completed the assessment (*M* = 21.8, *SD* = 16.8).

### 2.2 Measures

#### 2.2.1 Cortisol stress reactivity

At 22-years participants completed the TSST, a standardised paradigm involving a psychosocial stressor which has been demonstrated to elevate cortisol levels reliably (Kirschbaum et al., 1993). The TSST was delivered in standard format, including the preparation and execution of a 5-min speech followed by a 5-min spoken mental arithmetic task. The TSST took place in front of an unresponsive evaluative panel made up of two researchers unknown to the participants. Participants were instructed to talk about themselves during the speech following 5 min of preparation time. During the mental arithmetic task, participants were asked to count aloud backwards from 2023 to 0 in 17-step sequences. Following the stress phase of the experiment, participants were invited to rest for a period of 45-min. During the TSST, saliva samples were obtained through passive drooling into plastic containers at six times: at baseline, immediately post-test, and during the subsequent rest period (post + 10 min, post + 20, post + 30 and post + 45). In all cases, the TSST began at approximately 1500 h to reduce the effect of diurnal fluctuations on free cortisol (Allen et al., 2014), and participants refrained from eating or drinking for at least 30-min prior to the start of the protocol.

Saliva samples were kept at room temperature during the test session and then stored at −20 °C. Prior to assay, samples were thawed and then centrifuged at 3000 rpm for 5 min to produce clear supernatant fractions of low viscosity. Free cortisol was assayed by luminescence immunoassay (Immuno-Biological Laboratories, Hamburg, Germany). Inter- and intra-assay coefficients of variation were <7%. Cortisol concentrations are expressed as nmol/l.

#### 2.2.2 Offspring depression and anxiety

At 22-years offspring completed the 20-item, Centre for Epidemiological Studies Depression Scale (CESD) (Radloff, 1977), a well-established, self-report index of current depressive symptoms. Participants also completed the State-Trait Anxiety Inventory – Trait version (STAI-T), a reliable self-report measure of anxiety symptoms (Spielberger et al., 1983). In addition, at 8-years, 13-years and 16-years, diagnostic interviews were conducted using the Kiddie Schedule for Affective Disorders and Schizophrenia (Kaufman et al., 1997); and at 22-years using the Structured Clinical Interview for DSM-IV (SCID) (Spitzer et al., 1995). Both clinical interview schedules are widely used, have well established reliability, and include reporting of current and past disorders. Interviews were used to establish current and lifetime history of depressive and anxious disorders in offspring (present/absent).^2^ All interviews were conducted by trained clinical researchers and/or mental health professionals, and diagnoses were confirmed by clinical consensus by a panel of trained raters, blind to group.

#### 2.2.3 Life events

At 22-years offspring completed the Life Events Schedule (LES) (Goodyer et al., 2000), an interview measure of the occurrence of a range of significant undesirable life events and difficulties, which was used to index the occurrence of negative life events in the 12 months prior to assessment. Previous research using the same assessment has found associations between reported negative life events and the onset of depression (Goodyer et al., 2000). Consistent with previous reports, negative life events were operationalised as events that were rated by participants as scoring 4 (unpleasant) or above on a 5-point Likert scale from 0 (very pleasant) to 5 (very unpleasant), and as causing distress for 2 weeks or more.

#### 2.2.4 13-Year basal cortisol secretion

At age 13-years offspring collected saliva samples at 08:00 h and 20:00 h for 10 consecutive school days, following instructions supplied for home completion. We measured cortisol using enzyme-linked immunosorbent assay (ELISA) on 20 μl samples of saliva without extraction (antibody Cambio, Cambridge UK). Intra-assay variation was 4.1% and inter-assay variation was 7.6%. For each individual, we derived mean cortisol levels over the 10-day sampling period for morning and evening saliva collections. Previous analyses have identified elevations in mean morning cortisol to distinguish the PND group offspring from the control group (Halligan et al., 2004), and to predict subsequent offspring depressive symptoms (Halligan et al., 2007a). Such effects were not observed for evening cortisol. Therefore, the focus of the current analyses was on mean basal morning cortisol.

#### 2.2.5 Maternal depression

The presence and timing (on a month-by month basis since previous interview) of maternal depression at 22-years was assessed using the SCID. Detailed longitudinal information relating to maternal mental state was available as maternal clinical interviews were similarly conducted at all previous assessments. This was used to establish the overall duration of maternal depression, calculated as the total number of months of depression over the offspring's lifetime.

### 2.3 Statistical analysis

Before the main analysis, missing cortisol data (6% of 380 total saliva samples, due to insufficient saliva) were estimated in SPSS using the Expectation-Maximization (EM) algorithm (i.e., utilising the conditional expectation of the missing data, given the observed values and current estimates of the parameters), based on the other available cortisol samples. In addition, raw cortisol concentrations were checked for outliers (±3 *SD* from the mean) and for distributional properties. Four outliers were excluded from further analyses. Raw cortisol concentrations were log transformed to reduce positive skew (Pre-test: *Z* = 5.54; Post-test: *Z* = 4.14; Post + 10: *Z* = 2.51; Post + 20: *Z* = 4.11; Post + 30: *Z* = 3.38; Post + 45: *Z* = 4.94; all *ps* < .05).

HLM analyses were conducted in MLwiN 2.28 (Rasbash et al., 2009). HLM Group differences in cortisol reactivity over the TSST were analysed using two-level HLM. Within this approach, a level-1 model was estimated, representing individual variance in salivary cortisol concentrations over the course of the TSST (i.e., a main effect of time). Times were not constant and so were coded from the first spit onwards using decimals of the actual time in minutes (e.g. 0, .25, .35, .45, .55, .70). This produced the following model:

$$\text{Log}\;{\left(\text{cort}\right)}_{ij}=\pi_{0j}+\pi_{1j}{\left(\text{linear}\right)}_{ij}+\pi_{2j}{\left(\text{quadratic}\right)}_{ij}$$

In this model, the outcome was log transformed salivary cortisol concentrations, where *π*_0*ij*_ represented the intercept, *π*_1*ij*_ the linear slope and *π*_2*ij*_ the quadratic slope for cortisol reactivity over time.

A level-2 model was also estimated in which variance in the intercept and slope at level-1 were predicted by person-level differences that remain constant over the TSST. PND exposure was the main independent variable and the effects of gender, current depressive symptoms and history of depression in offspring were controlled for, given their established influence on cortisol reactivity in the TSST. This produced the following model, where the individual equations presented below refer to the main effect of exposure on the intercept of cortisol concentration (baseline) and the linear and quadratic slopes of cortisol concentrations over time (i.e., change from baseline), respectively, controlling for gender, current offspring depressive symptoms and history of depression in each:

$$\text{Intercept}\;\text{model}:\pi_{0j}=\beta_{00}+\beta_{01}{\left(\text{Exposure}\right)}_{j}+\beta_{02}{\left(\text{Gender}\right)}_{j}+\beta_{03}{\left(\text{CESD}\right)}_{j}+\beta_{04}{\left(\text{Lifetime}\;\text{Depr}\right)}_{j}+\zeta_{0j}$$

$$\text{Linear}\;\text{slope}\;\mathrm{mod}\text{el}:\pi_{1j}=\beta_{01}+\beta_{11}{\left(\text{Exposure}\right)}_{j}+\beta_{12}{\left(\text{Gender}\right)}_{j}+\beta_{12}{\left(\text{CESD}\right)}_{j}+\beta_{14}{\left(\text{Lifetime}\;\text{Depr}\right)}_{j}+\zeta_{1j}$$

$$\text{Quadratic}\;\text{slope}\;\text{model}:\pi_{2j}=\beta_{02}+\beta_{21}{\left(\text{Exposure}\right)}_{j}+\beta_{22}{\left(\text{Gender}\right)}_{j}+\beta_{23}{\left(\text{CESD}\right)}_{j}+\beta_{24}{\left(\text{Lifetime}\;\text{Depr}\right)}_{j}+\zeta_{2j}$$

We also explored the effects of key additional potential predictors of cortisol reactivity during the TSST by including them step-wise in a subsequent model as level-2 predictors. These variables were 13-year mean morning cortisol, total months of maternal depression over the course of the offspring's lifetime and the number of negative life events in the 12 months preceding the TSST. Finally, since anxiety disorders were also relatively prevalent in the sample (see Table 1), this model was then repeated, replacing lifetime depression and CESD scores with indices of lifetime anxiety diagnoses and STAI-T scores.^3^ This stepped approach to analyses was taken in consideration of the sample size in order to avoid overloading our models.

## 3 Results

### 3.1 Participant characteristics

Participants’ demographic and clinical characteristics are presented in Table 1. Participants in the PND and control group were similar in terms of socioeconomic background, age, gender, and concurrent depressive symptoms. However, a greater proportion of PND group offspring had experienced one or more episodes of depressive disorder; and there was a trend (*p* = .059) for PND group participants to report a greater number of significant negative life events in the 12-months prior to the TSST. As expected, PND group mothers also spent significantly more months depressed during their offspring's lifetime than their control group counterparts.

### 3.2 Variability in cortisol reactivity

The HLM for changes in cortisol concentrations over the course of the TSST yielded a significant positive linear fixed effect for time, *B* = .729, *SE* = .108, *χ*^2^(1) = 45.27, *p* < .001, and a significant negative quadratic fixed effect for time, *B* = .814, *SE* = .129, *χ*^2^(1) = 39.78, *p* < .01. There was also significant random variance in the intercept, *σ*_1_^2^ = .027, *SE* = .006, *χ*^2^(1) = 20.42, *p* < .001, which estimates pre-test baseline cortisol, and significant random variance in the linear, *σ*_1_^2^ = .572, *SE* = .148, *χ*^2^(1) = 14.94, *p* < .001, and quadratic slopes, *σ*_2_^2^ = .667, *SE* = .213, *χ*^2^(1) = 9.78, *p* < .005, of cortisol reactivity over time. There was therefore significant variation between participants in their intercepts and slopes for cortisol response, thereby supporting the modelling of individual differences in these parameters over time as a function of exposure to PND and our other key predictors.

### 3.3 Cortisol reactivity and maternal PND

**Figure 1:**
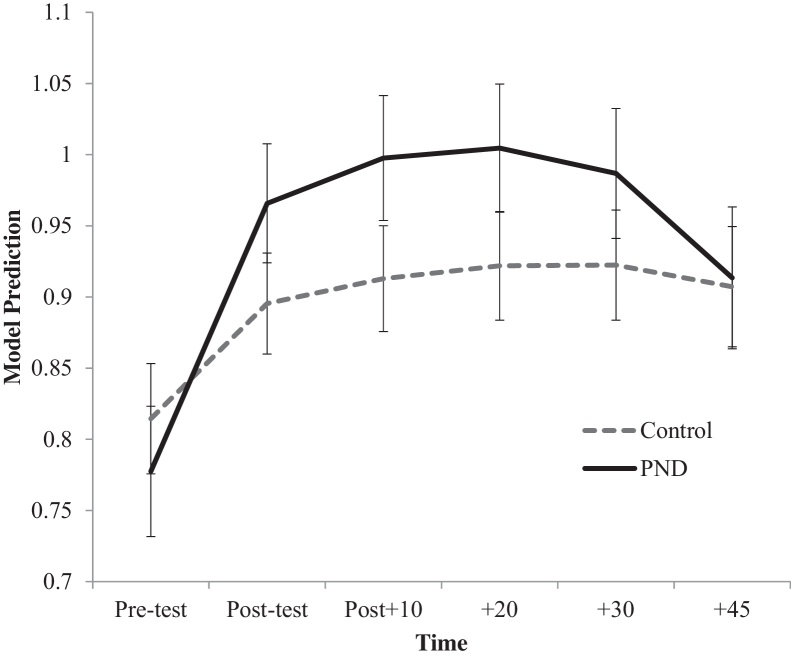
Hierarchical linear modelling Level-2 model predictions of changes in salivary cortisol responses across time during the Trier Social Stress Test; maternal postnatal depression versus control group participants. PND: postnatal depression; Error bars are one standard error.

We next modelled TSST cortisol response in relation to PND group status and key covariates, namely gender, lifetime history of depression, and concurrent depressive symptoms. Results are depicted in Fig. 1, with effects of each parameter on the intercept and the linear and quadratic slopes for cortisol presented in Table 2 (model 1). As can be seen, PND group status was not a significant predictor of the intercept, or in other words, baseline pre-test cortisol concentrations. However, the presence of maternal PND did predict steeper linear and quadratic slopes for cortisol reactivity over time (see Fig. 1 for model predicted cortisol levels across the TSST between groups; and Table 2 for model parameters). These effects were independent of the effect of gender, which itself was a significant predictor of the linear and quadratic slopes for cortisol reactivity over time, with males showing greater reactivity to the TSST than females (see Table 2). PND group effects were also independent of the effect of current depressive symptoms as measured by the CESD and lifetime history of depression (present/absent). Neither CESD scores nor lifetime history of depression significantly predicted any aspect of the model.

### 3.4 Potential intervening variables

To test the potential contribution to 22-year cortisol reactivity of pre-existing differences in basal cortisol at 13-years, intervening maternal depression and negative life events, a further model examined the extent to which these variables were significant predictors of cortisol responding over time, above and beyond the variance explained by PND. Each of these additional variables was inputted into the model in a step-wise fashion. First, offspring mean morning cortisol scores at 13-years were a significant predictor of the intercept (i.e., baseline pre-test cortisol responses), *B* = .040, *SE* = .020, *χ*^2^(1) = 4.07, *p* = .04, but there was no effect of 13-year mean morning cortisol levels on the linear, *B* = −.026, *SE* = .087, *χ*^2^(1) = .09, *p* = .76, or quadratic, *B* = .011, *SE* = 0.102, *χ*^2^(1) = .01, *p* = .92, slopes of cortisol reactivity over time. Next, the total months that mothers experienced depression showed no significant effects on the intercept, *B* = .000, *SE* = .002, *χ*^2^(1) = .00, *p* = 1.0, or linear, *B* = .008, *SE* = .009, *χ*^2^(1) = .77, *p* = .38, or quadratic, *B* = .008, *SE* = .011, *χ*^2^(1) = .56, *p* = .45, slopes. Finally, participants’ negative life events scores were entered into the model and this also showed no significant effects on the intercept, *B* = −.029, *SE* = .019, *χ*^2^(1) = 2.49, *p* = .11, or the linear, *B* = .033, *SE* = .083, *χ*^2^(1) = .16, *p* = .69, or quadratic, *B* = −.015, *SE* = .096, *χ*^2^(1) = .02, *p* = .89, slopes.

The resultant model is presented in Table 2 (model 2). In this model, with all variables included, exposure to PND was still a significant predictor of the linear and quadratic slopes for change in cortisol levels across the TSST. Gender also continued to be a significant predictor of the linear and quadratic slopes; and offspring 13-year mean morning cortisol scores continued to be a significant predictor of the intercept. Concurrent depressive symptoms, measured using the CESD scale, became a significant positive predictor of the intercept (see Table 2). There were no other significant effects (see Table 2 for statistics).

Finally, since anxiety disorders were also relatively prevalent in our sample, this last model was re-run replacing lifetime depression diagnoses and CESD scores with lifetime anxiety diagnoses and STAI-T scores respectively. Exposure to PND was still a significant predictor of the linear, *B* = .696, *SE* = .338, *χ*^2^(1) = 4.24, *p* < .05, and quadratic, *B* = −.834, *SE* = .391, *χ*^2^(1) = 4.55, *p* < .05, slopes of cortisol reactivity. As with CESD scores, STAI-C scores were a significant positive predictor of the intercept, *B* = .007, *SE* = .003, *χ*^2^(1) = 6.24, *p* < .05 but were not a significant predictor of the linear, *B* = −.013, *SE* = .013, *χ*^2^(1) = 1.00, *p* = .32, or quadratic, *B* = .015, *SE* = .015, *χ*^2^(1) = 1.10, *p* = .29, slopes. In contrast to this, lifetime anxiety diagnosis was a significant negative predictor of the intercept, *B* = −.179, *SE* = .056, *χ*^2^(1) = 10.135, *p* < .05. Lifetime anxiety diagnosis was not a significant predictor of the linear, *B* = −0.13, *SE* = .263, *χ*^2^(1) = .00, *p* = .96, or quadratic, *B* = .125, *SE* = .303, *χ*^2^(1) = .17, *p* = .68, cortisol reactivity slopes.

## 4 Discussion

We found that the offspring of PND mothers showed greater physiological reactivity to psychosocial challenge relative to offspring of mothers in our control group. This effect was independent of gender, current offspring depressive or anxious symptoms and lifetime history of depressive or anxiety disorder, pre-existing differences in baseline morning cortisol levels, the duration of subsequent episodes of maternal depression and recent negative life events. The main difference in response between the groups occurred at 10 min after stressor cessation, consistent with enhanced HPA reactivity to stress in the PND group. By the end of the testing period, cortisol levels in the PND group had returned to control group levels.

The current findings contribute to existing evidence concerning maternal PND, and parental depression more broadly, and its possible association with offspring HPA axis dysregulation (Azar et al., 2007; Dougherty et al., 2011; Waters et al., 2013), providing evidence of altered offspring sensitivity to social stress in adulthood. Elevated cortisol reactivity to stress has been found to have functional implications, for example, being associated with impairments to declarative memory (Kirschbaum et al., 1996). Previous research also suggests that profiles of elevated cortisol reactivity may be associated with depressive traits or negative affectivity (Gerra et al., 2001; Kunz-Ebrecht et al., 2003), and predict subsequent psychological disorder (Susman et al., 1997). More broadly, both elevated and blunted cortisol reactivity to stress are assumed to be maladaptive (Kunz-Ebrecht et al., 2003; Burke et al., 2005). Nonetheless, caution is needed in interpreting the current findings. PND group offspring showed a greater rise in cortisol in response to the stressor but their cortisol levels also showed stronger decline during the recovery period, relative to an overall shallower curve in the control group. Thus, the pattern of cortisol stress responding in the PND group is arguably more dynamic, and without external validation we cannot conclude that it is necessarily problematic.

We explored several potential contributors to TSST cortisol responding, in addition to maternal depression. Consistent with previous research, we found greater cortisol reactivity in males versus females in the current sample (Kirschbaum et al., 1992), and concurrent depressive and anxiety symptoms predicted elevated TSST cortisol baseline concentrations (Burke et al., 2005). Perhaps surprisingly, lifetime anxiety disorder was associated with lower TSST baseline cortisol. However, this finding is difficult to interpret given the mixture of diagnoses and current/past disorders included. We did not find evidence of an association between past history of depression and either cortisol intercept or slope during the TSST, and concurrent symptom levels were not associated with cortisol reactivity. The fact that depressive symptom levels were in the normal range and we had a modest number of cases in our sample may have limited our ability to detect such effects. However, as already noted, there remains a need for further investigation of the significance of the observed cortisol alterations.

Notably, we demonstrated also that elevations in basal morning cortisol levels at 13-years predicted higher overall TSST cortisol concentrations at 22-years. This evidence of stability in cortisol activity over almost a decade is consistent with previous findings which suggest that morning cortisol levels comprise stable, trait-like components, as well state driven effects which may be influenced by factors such as concurrent stress levels (Wust et al., 2000; Kunz-Ebrecht et al., 2004; Owens et al., 2014). Nonetheless, PND versus control group differences in cortisol reactivity to the TSST remained even once pre-existing group differences in 13-year basal morning cortisol levels were controlled for in analyses.

In principle, altered cortisol reactivity may be a consequence of numerous intervening biological processes, including factors operating within the HPA axis (e.g., increased adrenal sensitivity/enhanced cortisol release by the adrenal glands, or reduced negative feedback sensitivity operating via corticosteroid receptors), and suprahypothalamic influences (e.g. increased limbic input to the hypothalamus) (Gunnar and Quevedo, 2007). Research to date has typically focused on salivary cortisol secretion, which provides little insight into any underlying biological changes. Limited evidence based on responses to neurochemical stimulation of the HPA-axis suggests that either reduced glucocorticoid receptor negative feedback sensitivity or increased adrenal sensitivity may characterise high-risk probands (Holsboer et al., 1995; Ronsaville et al., 2006). Some studies also find evidence of altered neural activity in key components of the limbic system in the offspring of depressed parents, such as the amygdala (Monk et al., 2008; Joormann et al., 2012), but the implications of such observations for HPA activity remain to be established.

A number of causal factors might have contributed to altered cortisol stress reactivity observed in PND group offspring in our sample. There is evidence that environmental exposures influence offspring stress regulation, particularly in the perinatal period. Animal research has highlighted the potential for both maternal stressors during pregnancy and perturbations in caregiving postpartum to contribute to HPA axis dysregulation (Levine, 2005). We did not measure antenatal maternal depression in our sample, but it is likely to have been present in a proportion of PND group mothers, and previous research has linked antenatal affective disorder with increased basal cortisol secretion in offspring (Glover et al., 2010). As such, our findings may be explained, at least in part, by antenatal exposures. With regard to the postnatal environment, animal models highlight the sensitivity of HPA development to perturbations in the quality of early care (Weaver et al., 2004). Previous evidence from a subsample of the current study population showed that PND group mothers were more withdrawn or disengaged in interacting with their young infants, and that this pattern of parenting behaviour in the first postpartum year, but not at 5-years, predicted offspring basal cortisol secretion at 13-years (Murray et al., 2010). In the current report we found maternal PND to predict offspring cortisol reactivity at 22-years, even when controlling for subsequent maternal depression throughout the course of the study. This is consistent with animal models, and with some previous studies which have indicated that problems arising in the perinatal environment may be of particular importance to HPA axis stress responding (Brennan et al., 2008; Dougherty et al., 2011). At the same time, it is also highly likely that exposures later in development will be important. Although we took account of recent life events, the recurrence of maternal depression and offspring psychopathology, there are, of course, limits to the extent that any instrument can truly index the full range of environmental stressors. Persistent alterations in the quality of family life, ongoing perturbations in maternal or paternal mood and behaviour, stressors arising in the context of relationships within and outside of the home, and parental psychopathology other than depression, are among the many factors that may differentially affect the children of depressed parents (Goodman and Gotlib, 2001; Carter et al., 2001; Halligan et al., 2007b; Pawlby et al., 2011). Moreover, both depressive disorder and cortisol activity show a degree of heritability, and genetic influences are likely to have contributed to our findings, including in interaction with environmental factors (Alexander et al., 2009; Foley and Kirschbaum, 2010).

There are some major strengths of the current study, particularly the use of a well-characterised, longitudinally studied sample. Offspring psychological disorder was assessed at multiple time points and those with a history of disorder were retained in the sample, meaning that our sample is likely to represent high versus non-high risk probands relatively well. Nonetheless, there were some limitations, particularly the modest sample size and the restrictions this imposed on the number of covariates included in our models. As already discussed, the study design is essentially correlational and causal associations cannot be assumed; perturbations in the environment occurring in association with maternal depression, antenatal maternal mood disturbances, and co-occurring genetic vulnerabilities may all have contributed to the current findings. Although retention was extremely good considering study duration, there was somewhat greater attrition from the PND group versus the control group, and some evidence that participants with more chronically depressed mothers were particularly unlikely to attend. Consequently, those at greatest risk may be underrepresented in our sample. We note that similar problems are likely to be present in other samples in the field, but are less likely to be detected outside of a longitudinal study. Notwithstanding these limitations, our observations highlight a remarkably persistent association between maternal depression in the postnatal period and offspring adrenocortical reactivity to stress. Further investigation of the neurobiological underpinnings of the altered stress reactivity observed is indicated.

## Ethics

Procedures were approved by the University of Reading and the National Health Service Research Ethics Committees. Participants provided written informed consent prior to taking part.

## Role of the funding source

The research included has been funded by the UK Medical Research Council (grant G0701514 to SLH) and supported by the Tedworth Charitable Trust. These organisations had no involvement in the research beyond the provision of funding.

## Conflict of interest statement

The authors declare that there are no conflicts of interest.

## Figures and Tables

**Table 1:** Participant characteristics, reported by maternal postnatal depression status.

	PND *n* = 38	Control *n* = 38	Statistic
*Maternal characteristics*			
Proportion of middle class	63.2%	71.1%	*χ*^2^(1) = .14
Proportion separated from child's father	9.1%	20.6%	*χ*^2^(1) = 1.74
Total study months depressed, *M* (*SD*)	20.32 (14.52)	4.63 (4.56)	*t*(74) = −6.35***
*Offspring characteristics*			
Age, *M* (*SD*)	22.4 (0.6)	22.2 (0.7)	*t*(74) = −1.26
Proportion of males	50%	54.1%	*χ*^*2*^(1) = .12
Proportion with lifetime depression	47.4%	18.4%	*χ*^*2*^(1) = 7.21**
Proportion with lifetime anxiety disorder	39.5%	23.7%	*χ*^*2*^(1) = 2.19
Number of negative life events, *M* (*SD*)	1.3 (1.6)	0.7 (0.9)	*t*(74) = −1.92
13-year mean morning cortisol (ng/mL), *M* (*SD*)	3.3 (1.2)	2.6 (1.0)	*t*(70) = −2.59*
Proportion with current medication usage^a^	7.9%	10.5%	Fischer's *P* = 1.00
Depressive symptoms (CESD), *M* (*SD*)	10.8 (8.7)	10.7 (10.3)	*t*(73) = −.03
Anxiety symptoms (STAI-T), *M* (*SD*)	37.7 (10.9)	38.8 (11.5)	*t*(73) = .43

PND: postnatal depression; CESD: Centre for Epidemiological Studies Depression scale. STAI-T: State-Trait Anxiety Inventory, Trait form.

a Only medication known to influence cortisol activity is included: 1 participant from each group was taking antidepressants; 2 participants from each group were taking a contraceptive pill; and 1 control group participant was taking thyroid medication.

* *p* < .05.

** *p* < .01.

*** *p* < .005.

**Table 2:** Results of hierarchical linear modelling of cortisol responses to the Trier Social Stress Test.

Model	Model effects					
	Intercept		Linear		Quadratic	
	*B* (*SE*)	*χ*^2^ (df = 1)	*B* (*SE*)	*χ*^2^ (df = 1)	*B* (*SE*)	*χ*^2^ (df = 1)
*Model 1*						
Maternal PND^a^	−.011 (.045)	.06	.651 (.204)	10.20***	−.863 (.244)	12.52***
Gender^b^	−.041 (.044)	.88	−.611 (.200)	9.35***	.691 (.239)	8.34***
Depressive symptoms (CESD)	.004 (.002)	3.16	.009 (.011)	.63	.060 (.013)	.24
Lifetime depression^a^	−.068 (.050)	1.86	.073 (.229)	.10	.042 (.274)	0.02
*Model 2*						
Maternal PND^a^	−.019 (.060)	.10	.627 (.271)	5.36*	−.847 (.316)	7.18**
Gender^b^	−.066 (.045)	2.11	−.523 (.202)	6.71**	.560 (.235)	5.66*
Depressive symptoms (CESD)	.006 (.003)	4.53*	−.015 (.012)	1.58	.012 (.014)	.74
Lifetime depression	.050 (.050)	1.01	.028 (.224)	.02	−.053 (.260)	.04
13 year mean morning cortisol	.045 (.020)	5.10*	−.018 (.089)	.04	.001 (.105)	.00
Maternal total study months depression	.000 (.002)	.03	.009 (.009)	.86	−.008 (.011)	.58
Negative life events (LES)	−.029 (.019)	2.49	.033 (.083)	.02	−.051 (.096)	.02

PND: postnatal depression; CESD: Centre for Epidemiological Studies Depression Scale; LES: Life Events Schedule.

a 0 = absent, 1 = present.

b 1 = male, 2 = female.

* *p* < .05.

** *p* < .01.

*** *p* < .005.

## Footnotes

1 Reasons for attrition were as follows: lost from the study at previous time point (*n* = 5), could not be traced (*n* = 3), declined (*n* = 6), could not be scheduled (*n* = 2), unable to attend the University for assessments due to geographical/time constraints (*n* = 7), unwilling to complete laboratory tasks (*n* = 1).

2 At the 22-year follow-up, pre-screening ensured that any participants experiencing a current major depressive episode were not brought in for testing until their depression had remitted. This procedure did not result in the exclusion of any participants.

3 Other diagnoses were infrequent. Behavioural disorders were assessed at 8-years, 13-years and 16-years, resulting in diagnoses of attention deficit and hyperactivity disorder (*n* = 3), oppositional defiant disorder (*n* = 2) and conduct disorder (*n* = 1). Substance misuse was assessed in addition to depression/anxiety at 22-years, and one case of substance abuse detected.
